# Knowledge mapping and research trends of IL-33 from 2004 to 2022: a bibliometric analysis

**DOI:** 10.3389/fimmu.2023.1158323

**Published:** 2023-04-20

**Authors:** Jingyi Jin, Yantong Wan, Qiang Shu, Jinghua Liu, Dengming Lai

**Affiliations:** ^1^ Department of Neonatal Surgery, Children’s Hospital, Zhejiang University School of Medicine, National Clinical Research Center for Child Health, Hangzhou, China; ^2^ Guangdong Provincial Key Laboratory of Proteomics, Department of Pathophysiology, School of Basic Medical Sciences, Southern Medical University, Guangzhou, China; ^3^ Department of Thoracic and Cardiovascular Surgery, Children’s Hospital, Zhejiang University School of Medicine, National Clinical Research Center for Child Health, Hangzhou, China

**Keywords:** IL-33, immunity, bibliometric analysis, VOSviewer, CiteSpace

## Abstract

**Background:**

IL-33 has been studied widely but its comprehensive and systematic bibliometric analysis is yet available. The present study is to summarize the research progress of IL-33 through bibliometric analysis.

**Methods:**

The publications related to IL-33 were identified and selected from the Web of Science Core Collection (WoSCC) database on 7 December 2022. The downloaded data was analyzed with bibliometric package in R software. CiteSpace and VOSviewer were used to conduct IL-33 bibliometric and knowledge mapping analysis.

**Results:**

From 1 January 2004 to 7 December 2022, 4711 articles on IL-33 research published in 1009 academic journals by 24652 authors in 483 institutions from 89 countries were identified. The number of articles had grown steadily over this period. The United States of America(USA) and China are the major contributors in the field of research while University of Tokyo and University of Glasgow are the most active institutions. The most prolific journal is Frontiers in Immunology, while the Journal of Immunity is the top 1 co-cited journal. Andrew N. J. Mckenzie published the most significant number of articles and Jochen Schmitz was co-cited most. The major fields of these publications are immunology, cell biology, and biochemistry & molecular biology. After analysis, the high-frequency keywords of IL-33 research related to molecular biology (sST2, IL-1), immunological effects (type 2 immunity, Th2 cells), and diseases (asthma, cancer, cardiovascular diseases). Among these, the involvement of IL-33 in the regulation of type 2 inflammation has strong research potential and is a current research hotspot.

**Conclusion:**

The present study quantifies and identifies the current research status and trends of IL-33 using bibliometric and knowledge mapping analysis. This study may offer the direction of IL-33-related research for scholars.

## 1 Introduction

IL-33 belongs to the IL-1 family and was once called NF-HEV and DVS 27. DVS-27 was found to be a canine protein encoded by an unknown mRNA in 1999 (1). In 2003, a human nuclear protein was found to be highly expressed in endothelial cells from lymphoid organs and associated with chromatin, which is strongly similar to DVS27 (2). In 2005, it was discovered that the human HF-HEV protein has a similar structure to the IL-1 family cytokines. According to these discoveries, the name IL-33 was then named (3). IL-33 is a tissue-derived nuclear cytokine that is highly expressed in the nuclei of numerous cell types, including endothelial, epithelial, and fibroblast-like cells (4). By attaching to a heterodimer made of its specific receptor ST2 and IL-1 receptor accessory protein (IL-1RAcP), IL-33 activates the NF-kB and MAPK cellular signaling pathways to activate cells (5).

It was demonstrated that IL-33 is an alarm signal generated in the extracellular space following cellular injury (4, 6, 7). The primary target cells for IL-33 are tissue-resident immune cells such as type 2 innate lymphocytes (ILC2) (8, 9). Once IL-33 stimulated ILC2 through ST2 receptor, they then secrete large amounts of Th2 cytokines, particularly IL-5 and IL-13 (10–12). ST2, the specific receptor for IL-33, is constitutively expressed on mast cells and Th2 immune cells and is involved in type 2 immune responses (13, 14). In addition, ST2 can be inducibly expressed on Th1 immune cells such as CD8^+^ T cells, NK cells, and NKT cells, and regulate type 1 immune responses in infections and chronic inflammation (15, 16). Moreover, previous studies have shown a direct association between IL-33 and inflammatory diseases such as asthma (17), inflammatory bowel disease(IBD) (18), chronic obstructive pulmonary disease(COPD) (19), myocardial infarction (20, 21) and atopic dermatitis (22). Also, IL-33, a pleiotropic cytokine, is closely associated with cancer such as colorectal cancer (23) and myeloproliferative neoplasms (24), which may be related to mast cells and tumor microenvironment(TME).

IL-33 is a rapidly growing and popular field of research, with the number of studies and articles related to IL-33 increasing over the last 20 years. IL-33 reviews have been published from various perspectives (25, 26). Nevertheless, there is currently no comprehensively integrated analysis of the authoritative authors and institutions, research progress and emerging trends related to IL-33.

Bibliometric analysis is the qualitative and quantitative analysis of literature studies using mathematical and statistical methods (27, 28). Bibliometrics can provide a comprehensive analysis of the countries, institutions, authors, and journals of the selected articles in terms of their contribution to this research field (29). In addition, it can assess the possible trends and emerging hotspots in this field (30). In the present study, we utilize CiteSpace and VOSviewer for bibliometric and visual analysis to construct a knowledge map of relevant scientific research, to sort out and analyze the development trends and emerging hotspots of IL-33 research, and to provide future research perspectives.

## 2 Materials and methods

### 2.1 Data collection

We collected data from Web of Science Core Collection (WoSSC) bibliographic collection, frequently used in bibliometrics, which is currently one of the biggest and most extensive electronic scientific literature database in the world (30). The Data were systematically retrieved between 1 January 2004 and 7 December 2022 and downloaded from the WoSCC database on 22 August 2022 to avoid bias. The following search formula utilized in this research was set as follows: (“IL-33” OR “IL33” OR “interleukin 33” OR “NF-HEV” OR “nuclear factor from high endothelial venules” OR “IL1F11” OR “interleukin-33” OR “c9orf26” OR “dvs27” OR “Nuclear Factor For High Endothelial Venules” OR “Interleukin-1 Family Member 11” OR “dvs27-Related Protein” OR “Interleukin-33” OR “DKFZp586H0523” OR “Chromosome 9 Open Reading Frame 26 (NF-HEV)” OR “Interleukin-1 Family, Member 11” OR “IL-1F11” OR “NFHEV” OR “NFEHEV”). The only available publication types were Article and Review, and the language was English only. Additionally, the research findings were documented with the content of “Full Record and Cited Reference” in the “Plain Text” format. Finally, 4711 original articles or reviews were included.

### 2.2 Data analysis

The downloaded files were imported into CiteSpace 6.1.R3, VOSviewer 1.6.18, and Microsoft Excel 2019 to conduct the bibliometric and knowledge mapping analysis. Before the keyword co-occurrence analysis, synonyms were merged into one word, nonsense words were deleted, and identical authors and institutions with different spellings were merged.

CiteSpace, developed by Prof. Chaomei Chen, is a universally used program based on JAVA for bibliometric and visual analysis (31). CiteSpace can uncover potential information in the vast literature through visualization, detecting national and institutional contributions and collaborations, disciplinary distribution, citation and co-citation counts, research hotspots, and more.

VOSviewer is another bibliometric analysis software developed by Nees Jan van Eck and Ludo Waltman for building and viewing bibliometric maps based on web data, from which key information from numerous publications can be exacted (32). It can be used to create visual network maps based on collaborative data or keyword maps based on co-occurrence data. VOSviewer’s main goal is to give users a thorough grasp of the dynamics and structure of scientific research.

## 3 Results

### 3.1 Annual publication growth trend

**Figure 1:**
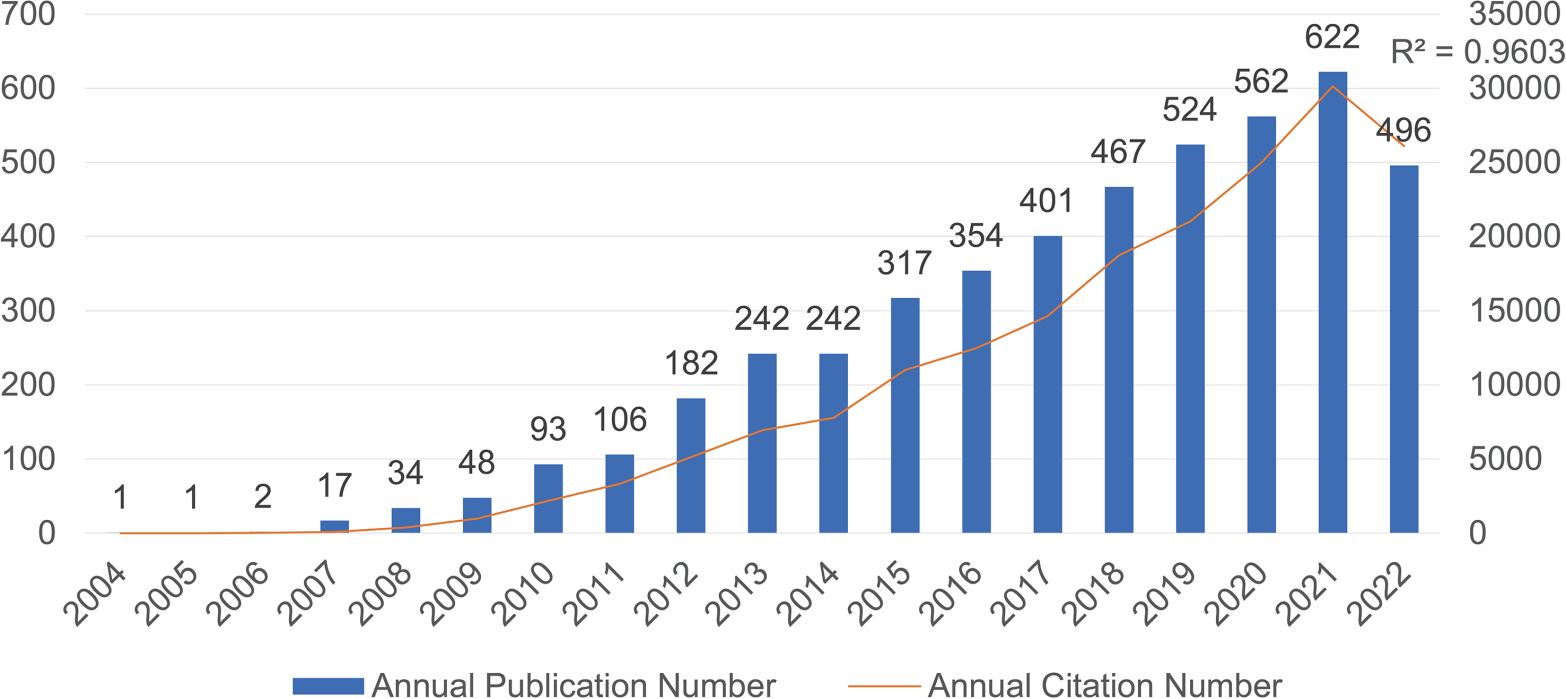
The number of annual publications and citations of IL-33 research from 2004 to 2022 has been steadily increasing.

According to our research strategy, there are a total of 4,711 publications related to IL-33 obtained from the WoSCC database from 2004 to 2022. The annual publication number and citation number with IL-33 are shown in **Figure 1**. In 2004-2006, only 1-2 articles were published each year. However, from 2007 to 2022, the number of publications increased steadily, with a slight stagnation in 2014, and the correlation coefficient R^2^ is 0.9603. Besides, the upward trend in publication citation frequency from 2004 to 2022 was also calculated (**Figure 1**). The steady increase in Np (number of publications) and Nc (number of citations) indicates that IL-33 continues to be attractive for more and more scholars to conduct research related to IL-33 and that this field needs more prospective studies in the future.

### 3.2 Analysis of countries and institutions

A total of 89 countries and 483 institutions were involved in IL-33-related research. The top 10 most prolific countries in IL-33 publications based on Np and Nc were ranked (**Table 1**). The leading countries are the USA (1,354) and China (1,172), both exceeding 1,000 publications, followed by Japan (515) and all other countries with less than 500 publications.

**Table 1:** The top 10 productive countries with publications concerning IL-33.

Rank	Countries	Np	Countries	Nc	Countries	Total Link Strength
1	USA	1354	USA	81160	USA	910
2	China	1172	England	26369	England	490
3	Japan	515	China	20774	Germany	393
4	England	323	Japan	18387	China	377
5	Germany	303	Germany	15937	France	278
6	France	238	France	14494	Netherlands	243
7	Italy	230	Netherlands	13432	Australia	218
8	South Korea	209	Scotland	12185	Switzerland	211
9	Canada	159	Switzerland	9843	Scotland	206
10	Netherlands	152	Italy	9632	Italy	193

**Figure 2:**
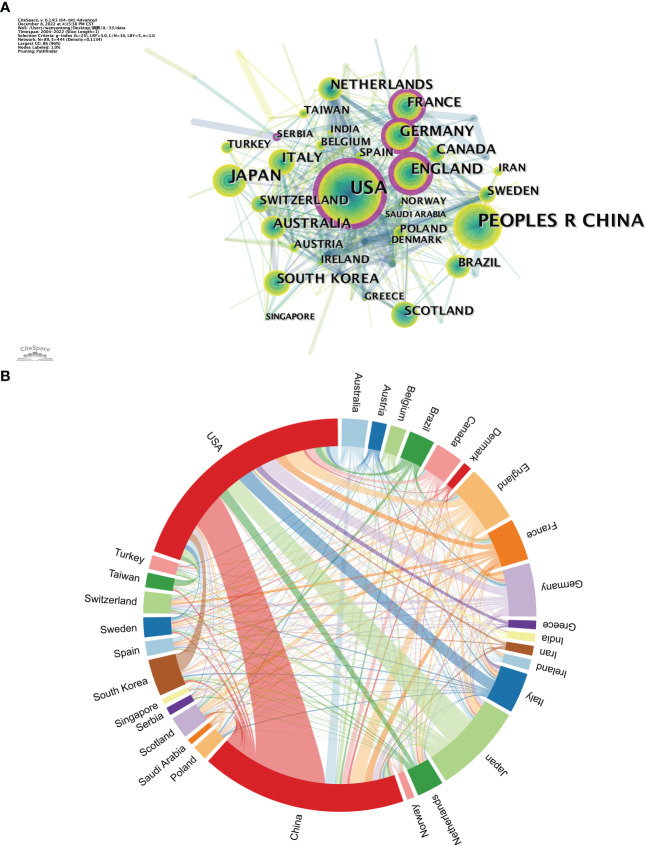
The analysis of countries related to IL-33. **(A)** The co-occurrence map of countries. The node size means the co-occurrence frequencies, while the linkages mean the co-occurrence relationships. Nodes with purple round mean high betweenness centrality (≥0.1). **(B)** The network map of cooperation between countries.

It is worth noting that while both China and the USA contribute nearly a quarter of the publications in the IL-33 field, the USA had a total of 811,160 citations, four times of China (26,369), indicating that the USA was the most influential country in this field in terms of both quantity and quality of articles so far. In addition, the close cooperation between the various countries is shown in **Figures 2A**, **2B**. The purple-round nodes indicate high betweenness centrality (≥0.1). The top five countries with high centrality are the USA, England, France, Germany, and Serbia (**Figure 2A**). The country’s co-occurrence density is 0.11, showing active cooperation among them. The closest cooperation occurs between the USA and China, followed by the USA and Japan (**Figure 2B**).

**Figure 3:**
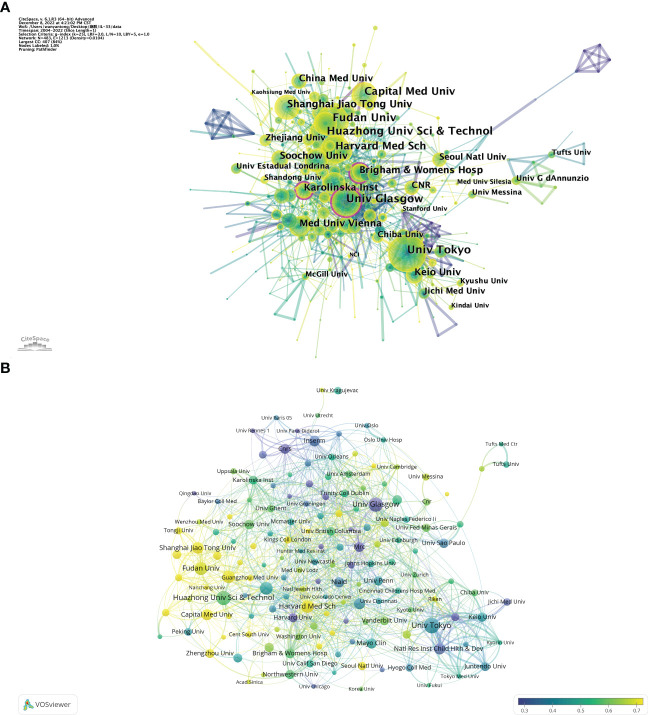
The analysis of research institutions related to IL-33. **(A)** The co-occurrence map of research institutions. The node size means the co-occurrence frequencies, while the linkages mean the co-occurrence relationships. Nodes with purple round mean high betweenness centrality (≥0.1). **(B)** The proportion of institutional publications to complete publications during the last five years. When the color bias is towards yellow, the ratio is larger; when the color bias is towards purple, the ratio is lower. VosViewer.

The top 10 science research institutions regarding the number of publications and frequency of citations were the University of Tokyo from Japan contributes the most publications (Np: 92), followed by Huazhong University of Science (Np: 84) from China and the University of Glasgow (Np: 81) from England (**Table 2**). Also, the University of Tokyo and the University of Glasgow were the top 10 institutions in terms of Nc, reflecting their scientific strength and importance. The intensive cooperation among institutions of the University of Glasgow, Brigham and Women’s Hospital, and Karolinska Institute had high centrality (**Figure 3A**), suggesting that these institutions were significant in the IL-33 research. **Figure 3B** depicts the proportion of institutional publications relative to complete publications during the last five years. The findings demonstrate a significant increase in the number of studies carried out during the previous 5 years by Harvard Medical School, Shanghai Jiao Tong University, Guangzhou Medical University, and other organizations. In comparison, the University of Glasgow, Medical Research Council, Center for Child Health & Development, Centre National De La Recherche Scientifique(CNRS), and other institutions have undertaken relatively few studies over the last 5 years.

**Table 2:** The top 10 productive research institutions with publications concerning IL-33.

Rank	Institution	Np	Country	Institution	Nc	Country
1	Univ Tokyo	92	Japan	MRC	9175	England
2	Huazhong Univ Sci & Technol	84	China	Univ Glasgow	8920	England
3	Univ Glasgow	81	England	Univ Calif San Francisco	6166	USA
4	Fudan Univ	75	China	Harvard Univ	5702	USA
5	Harvard Med Sch	72	USA	Univ Penn	4910	USA
6	Univ Pittsburgh	69	USA	Univ Tokyo	4751	Japan
7	Sun Yat Sen Univ	67	China	NIAID	4683	USA
8	Shanghai Jiao Tong Univ	63	China	CRNS	4440	France
9	Capital Med Univ	62	China	Keio Univ	4175	Japan
10	Natl Res Inst Child Hlth & Dev	57	Japan	Univ Toulouse	4087	France

### 3.3 Analysis of journal

**Figure 4:**
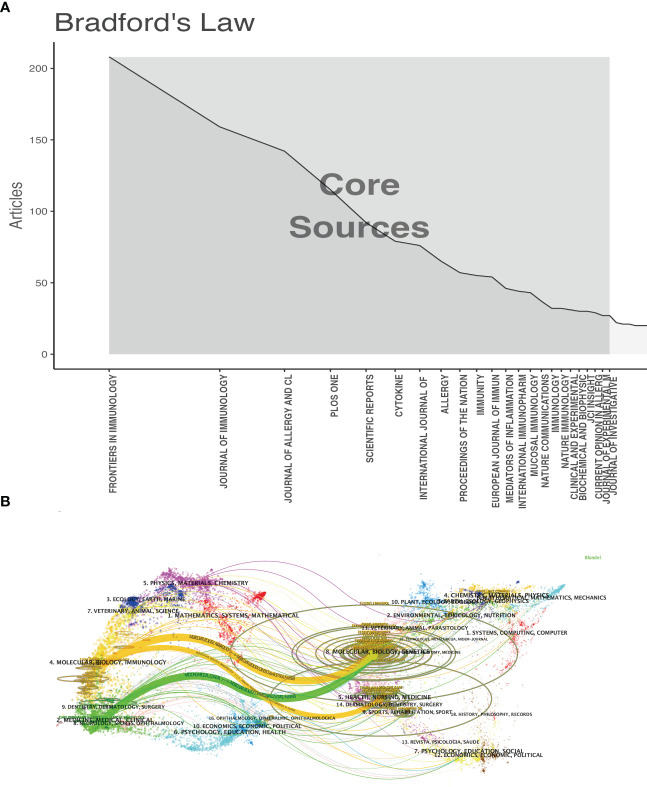
The analysis of academic journals related to IL-33. **(A)** Bradford’s Law according to the academic journals. **(B)** A dual-map overlay of the journals on IL-33 research. Clusters of citing journals are on the left, cited journals are on the right, and colored trails between them indicate the cited relationships.

A visual analysis of published journals and co-cited journals to show active and influential journals about IL-33 was performed. We discovered that 4,711 IL-33-related publications were published in 1009 academic journals. **Table 3** and **Figure 4A** show that the journal with the most publications is Frontier of Immunology (208, 4.42%), followed by Journal of Immunology (159, 3.38%), Journal of Allergy and Clinical Immunology (142, 3.01%) and PLOS ONE (115, 2.44%). Furthermore, five are in the Q1 JCR division, and seven have an impact factor (IF) of over 5 among the top 10 journals. Seven of the top ten co-cited academic journals have been cited over 5,000 times. The journals with the highest citations are the Journal of Immunology (15,704) and the Journal of Allergy and Clinical Immunology (12,281). Five of the top ten co-cited journals are in the Q1 JCR, and six have an impact factor of over 10.

**Table 3:** The top 10 productive academic journals with publications concerning IL-33.

Rank	Journal	Np	% of (4711)	IF(JCR2021)	JCR quatile	Co-Cited Journal	Nc	IF(JCR2020)	JCR quatile
1	Frontiers In Immunology	208	4.42%	8.786	Q1	J Immunol	15704	5.446	Q2
2	Journal Of Immunology	159	3.38%	5.446	Q2	J Allergy Clin Immun	12281	14.29	Q1
3	Journal Of Allergy And Clinical Immunology	142	3.01%	14.29	Q1	Immunology	9926	7.215	Q2
4	Plos One	115	2.44%	3.752	Q2	P Natl Acad Sci Usa	8473	12.779	Q1
5	Scientific Reports	92	1.95%	4.996	Q2	Nat Immunol	6383	31.25	Q1
6	Immunology	79	1.68%	7.215	Q2	J Exp Med	6342	17.579	Q1
7	International Journal Of Molecular Sciences	76	1.61%	6.208	Q1	Nature	5505	69.504	Q1
8	Cytokines	65	1.38%	3.926	Q3	Plos One	4589	3.752	Q2
9	Allergy	57	1.21%	14.71	Q1	J Biol Chem	3685	5.486	Q2
10	Proceedings Of The National Academy Of Sciences Of The United States Of America	55	1.17%	12.779	Q1	Nat Rev Immunol	3662	108.555	Q2

**Figure 4B** is a dual-map overlay of journals, which can provide a more visual representation of the distribution of individual academic journals, the development of citation trajectories, and the change in research focus. Overall, **Figure 4B** shows that Molecular/Biology/Genetics journals, as well as Molecular/Biology/Immunology journals, frequently cite articles published in Molecular/Biology/Genetics journals.

### 3.4 Analysis of authors

A total of 24,652 authors participated in the research of IL-33. **Table 4** shows the top 10 most prolific and the top 10 most cited authors in IL-33 study. We can see that Andrew N. J. McKenzie from Medical Research Council, England led with 58 articles and 10,769 citations, followed by Susumu Nakae from University of Tokyo, Japan, with 48 articles but ninth in citations (3,552).

**Table 4:** The top 10 productive authors with publications and citation frequency concerning IL-33.

Rank	Author	Np	Country	Author	Nc	Country
1	Mckenzie, Andrew N. J.	59	England	Mckenzie, Andrew N. J.	10769	England
2	Nakae, Susumu	50	Japan	Liew, Foo Yew	7472	England
3	Liew, Foo Yew	44	England	Girard, Jean-Philippe	6055	France
4	Saito, Hirohisa	42	Japan	Fallon, Padraic G.	4648	Ireland
5	Kita, Hirohito	37	USA	Xu, Damo	4646	China
6	Girard, Jean-Philippe	36	France	Artis, David	4350	USA
7	Matsumoto, Kenji	33	Japan	Locksley, Richard M.	3801	USA
8	Ryffel, Bernhard	27	France	Mcinnes, Iain B.	3580	England
9	Verri, Waldiceu A., Jr.	25	Brazil	Nakae, Susumu	3552	Japan
10	Xu, Damo	25	China	Dinarello, Charles Anthony	3545	USA

**Figure 5:**
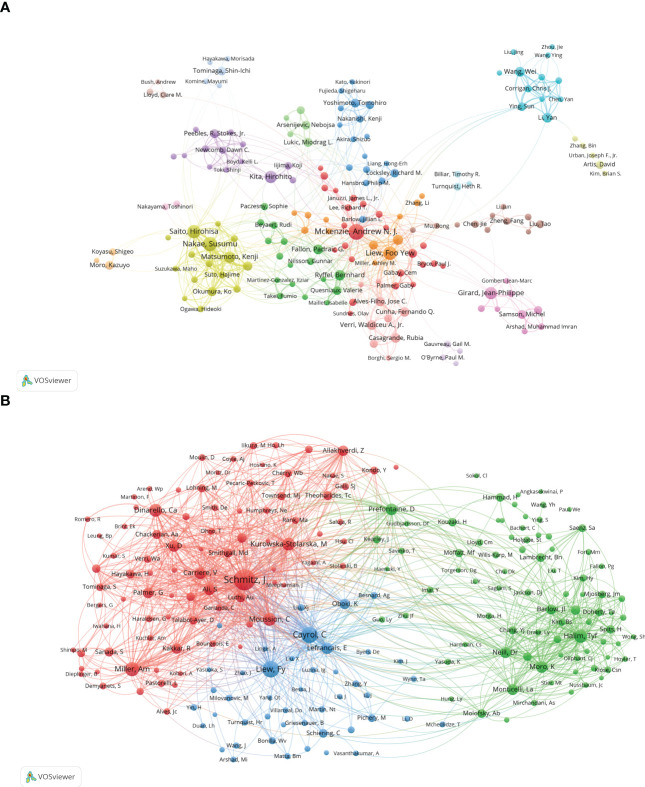
The analysis of authors related to IL-33. **(A)** The co-occurrence authors’ map of IL-33 research. The varied colored nodes reflect the authors in various clusters. The node size means the co-occurrence frequencies, while the linkages mean the co-occurrence relationships between authors. **(B)** The co-cited authors’ map of IL-33 research. The node size means the frequency of their occurrence. VosViewer.

**Figure 5A** shows a cooperation network between authors, providing expert information for finding research partners. The 18 colors represent 18 clusters in **Figure 5A**. Andrew N. J. McKenzie and Foo Yew Liew are at the heart of the collaborative network. Authors collaborate actively in conducting the IL-33 study, especially among authors in the same cluster, e.g., Susumu Nakae and Hirohisa Saito. Close collaboration could also be observed between authors in different clusters, e.g., Andrew N. J. McKenzie and Foo Yew Liew.

The co-citation of authors refers to when different authors are cited by another articles at the same time, these authors form a co-citation relationship. When the number of co-citations is higher, their academic research is more similar, and the analysis reflects their research strength (**Figure 5B**). The authors were mainly divided into 3 clusters: Jochen Schmitz, Virginie Carriere, etc.(red); Foo Yew Liew, Corinne Cayrol, etc.(blue); Timotheus Y F Halim, Daniel R Neill, etc.(green).

### 3.5 Analysis of reference

**Table 5** shows the top 15 most cited articles about IL-33, with the top 7 articles having more than 1000 citations. The article, which was most cited, was Jochen Schmitz’s “IL-33, an interleukin-1-like cytokine that signals *via* the IL-1 receptor-related protein ST2 and induces T helper type 2-associated cytokines”, published in Immunity in 2005 with 2628 citations. In this article, IL-33 was introduced firstly (3), which set the stage for subsequent research on IL-33. Daniel R Neill’s “Nuocytes represent a new innate effector leukocyte that mediates type-2 immunity” in Nature in 2010 with 1507 citations (11).

**Table 5:** The top 10 co-cited references concerning IL-33.

Rank	Title	First Author	Journal	Nc	Year
1	IL-33, an interleukin-1-like cytokine that signals *via* the IL-1 receptor-related protein ST2 and induces T helper type 2-associated cytokines	Schmitz, J	IMMUNITY	2628	2005
2	Nuocytes represent a new innate effector leukocyte that mediates type-2 immunity	Neill, Daniel R.	NATURE	1507	2010
3	Macrophage plasticity and polarization in tissue repair and remodelling	Mantovani, Alberto	JOURNAL OF PATHOLOGY	1444	2013
4	A Large-Scale, Consortium-Based Genomewide Association Study of Asthma	Moffatt, Miriam F.	NEW ENGLAND JOURNAL OF MEDICINE	1400	2010
5	Innate production of T(H)2 cytokines by adipose tissue-associated c-Kit(+)Sca-1(+) lymphoid cells	Moro, Kazuyo	NATURE	1398	2010
6	The Interleukin-1 Family: Back to the Future	Garlanda, Cecilia	IMMUNITY	1205	2013
7	Innate lymphoid cells promote lung-tissue homeostasis after infection with influenza virus	Monticelli, Laurel A.	NATURE IMMUNOLOGY	1060	2011
8	The IL-1-Like Cytokine IL-33 Is Constitutively Expressed in the Nucleus of Endothelial Cells and Epithelial Cells *In Vivo*: A Novel ‘Alarmin’?	Moussion, Christine	PLOS ONE	862	2008
9	Human IL-25-and IL-33-responsive type 2 innate lymphoid cells are defined by expression of CRTH2 and CD161	Mjosberg, Jenny M.	NATURE IMMUNOLOGY	849	2011
10	Systemically dispersed innate IL-13-expressing cells in type 2 immunity	Price, April E.	PROCEEDINGS OF THE NATIONAL ACADEMY OF SCIENCES OF THE UNITED STATES OF AMERICA	831	2010
11	House dust mite allergen induces asthma *via* Toll-like receptor 4 triggering of airway structural cells	Hammad, Hamida	NATURE MEDICINE	813	2009
12	Disease-associated functions of IL-33: the new kid in the IL-1 family	Liew, Foo Y.	NATURE REVIEWS IMMUNOLOGY	757	2010
13	IL-33, the IL-1-like cytokine ligand for ST2 receptor, is a chromatin-associated nuclear factor *in vivo*	Carriere, Virginie	PROCEEDINGS OF THE NATIONAL ACADEMY OF SCIENCES OF THE UNITED STATES OF AMERICA	742	2007
14	IL-33 and ST2 comprise a critical biomechanically induced and card ioprotective signaling system	Sanada, Shoji	JOURNAL OF CLINICAL INVESTIGATION	729	2007
15	Overview of the IL-1 family in innate inflammation and acquired immunity	Dinarello, Charles A.	IMMUNOLOGICAL REVIEWS	710	2018

**Figure 6:**
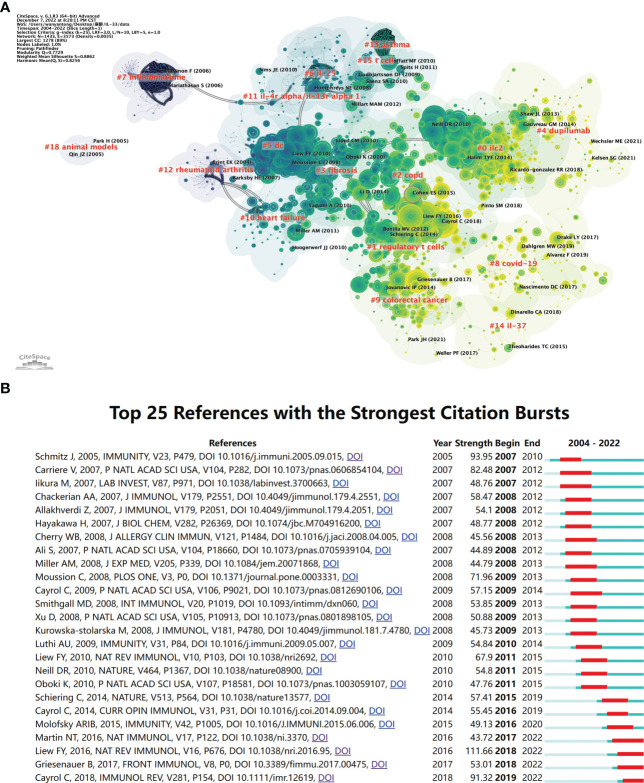
The analysis of references related to IL-33. **(A)** Clustering of references based on the similarity between references, including #0 ilc2, #1 regulatory t cells, #2 copd, #3 fibrosis, #4 dupilumab, #5 dc, #6 il-25, #7 inflammasome, and so on. **(B)** The top 25 references with strong citation bursts. A red bar means high citations in the corresponding year. CiteSpace.

The knowledge structure of the research field can be objectively displayed using the co-citations cluster analysis. To further depict the groups of references that were co-cited, we created a network map (**Figure 6A**). “The Oxidation of the alarmin IL-33 regulates ST2-dependent inflammation” published by E Suzanne Cohen in 2015 and had a high centrality (33), which proposed a novel mechanism for regulating IL-33, namely oxidation-driven conformational changes, unlike the previous restriction of IL-33 activity through caspase (6, 34, 35) or soluble ST2 and IL1RAcP (20, 36). The degree of correlation between the articles, classified into 18 categories, serves as the foundation for cluster classification. The largest cluster is #0 ilc2. The earliest clusters to begin research were #18 animal models, #7 inflammasome, and #12 rheumatoid arthritis. Later research developed into #11 il-4r alpha/il-13r alpha 1, #10 heart failure, and #5 dc. #13 asthma is relatively closely linked to #15 t cells and #2 copd to #1 regulatory t cells. In recent years the closeness of the links between the study areas has decreased, with #4 dupilumab, #8 covid-19, #14 il-37, and #9 colorectal cancer becoming more independent clusters.

References with citation bursts are those whose citations significantly and suddenly increase over a certain period. We listed the top 25 with the strongest citation bursts in **Figure 6B**. The earliest three citation burst began in 2007. The strongest burst (strength=111.66) occurred in a paper entitled “Interleukin-33 in health and disease” (26), published in Nature Reviews Immunology by Foo Yew Liew et al. in 2016, with citation burst from 2018 to the present. “IL-33, an interleukin-1-like cytokine that signals *via* the IL-1 receptor-related protein ST2 and induces T helper type 2-associated cytokines” by Jochen Schmitz, published in Immunity in 2005, also had a high burst (Strength=93.95) (3). According to the findings, 2018 had the highest citation bursts, followed by 2009, indicating that the high-burst articles in these two years lead to a research boom. Notably, four references are still in the burst.

### 3.6 Analysis of hotspots and frontiers

**Figure 7:**
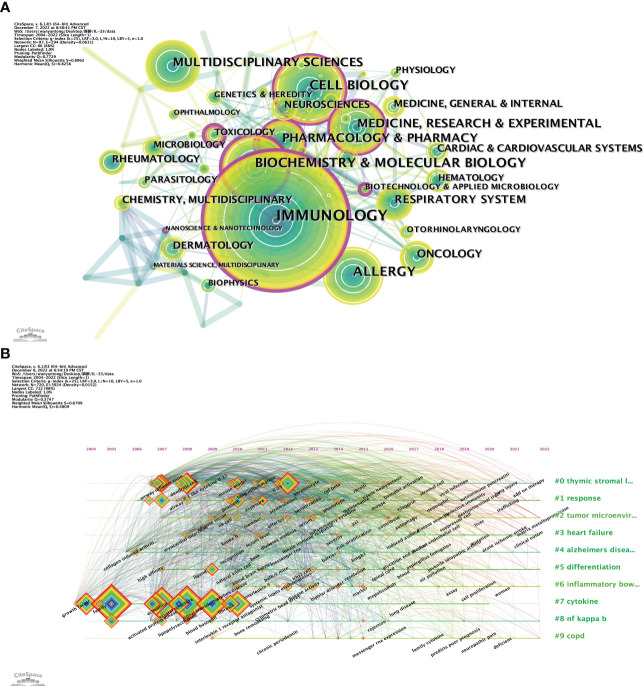
The analysis of research fields. **(A)** and keywords concerning IL-33 **(B)**. **(A)** The co-occurrence map of research fields. **(B)** A timeline view of keywords. CiteSpace.

Our study includes 97 research areas related to IL-33 (**Figure 7A**), with current research focusing on immunological, biochemistry & molecular biology, and cell biology. It can be seen that IL-33 has been researched in broad directions, involving research in several fields.

Usually, the keywords reflect the topic and research content of the articles. We can quickly comprehend the focus and trend of research in a particular field by analyzing the co-occurrence of keywords. We list the top 20 most common keyword about IL-33. In addition to IL-33, inflammation (560), asthma (479), cytokines (409), sST2 (381), ILCs (277), and allergic inflammation (248) are the keywords that appeared more than 200 times in the study and have more than 50 total link strength. These 20 keywords mainly focus on the biology and function in disease of IL-33.

The timeline viewer of keywords helps to analyze the evolution of these keywords in diverse clusters. In **Figure 7B**, we can visualize the progression of keywords in the IL-33 field and the keywords that are the research focus in each phase. Nine of the 10 clusters (except #8 cytokine) are still in progress. #0 thymic stromal lymphopoietin (TLPS) is the largest cluster, and the first keywords to appear in the field were airway inflammation, dendritic cell, and IL-1-like cytokine IL-33. At the same time, add-on therapy and lung injury are the latest research directions. #8 cytokine was the first cluster to be studied and the most cited outbreak, but it will no longer be a research priority after 2020. #6 IBD is the latest cluster, with the main keywords being systemic lupus erythematosus, disease activity, and bipolar disorder. The evolution of research keywords reflects the early nascent phase focused on studying IL-33 molecular biology and related mechanisms, and now focuses more on exploring the mechanisms of occurrence and applications in various diseases.

**Figure 8:**
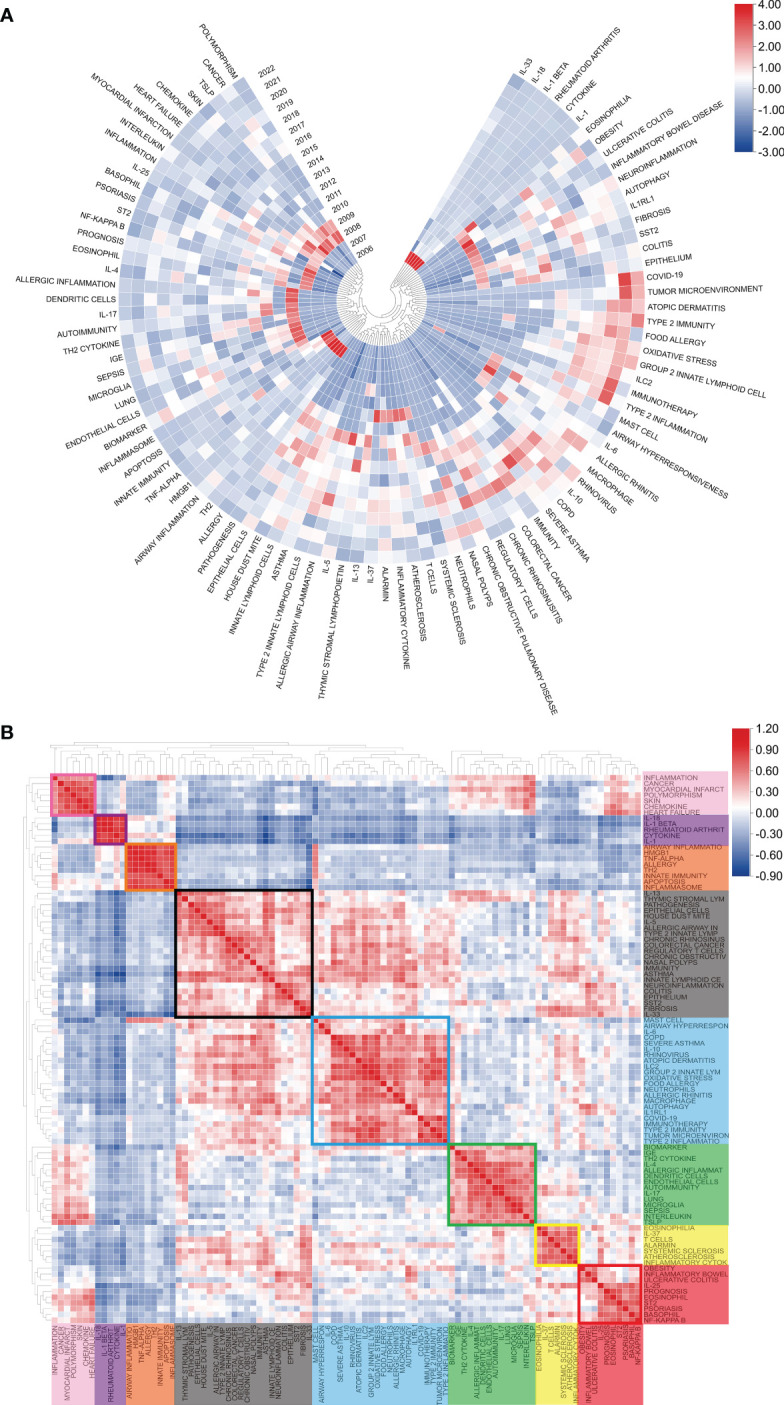
Heatmap analysis of IL-33 keywords. **(A)** The annual heatmap related to IL-33 research. The annual popularity of the keyword is measured as the ratio of the number of citations to the total citations in the same year. **(B)** Keyword relevance heatmap of IL-33. Keywords with high popularity in the same period are grouped together in a category and denoted by distinct colors.

**Figure 8A** displays the annual popularity of the keyword related to IL-33 research from 2004 to 2022, which is measured as the ratio of the number of citations to the total citations in the same year. In recent years, keywords like COVID-19, group 2 innate lymphoid cells, type 2 immunity, and tumor microenvironment have had relatively significant annual popularity, indicating that these terms have become a burgeoning research hotspot. In contrast, keywords like basophil and allergy have recently had relatively low annual popularity. The popularity correlation of keywords is shown in **Figure 8B**. Keywords with high popularity in the same period are grouped together in a category and denoted by distinct colors. There are 8 clusters: the pink cluster (inflammation, cancer, myocardial infarct, etc.), purple cluster (IL-18, IL-1β, rheumatoid arthrit, etc.), orange cluster (airway inflammation, TNF-α, allergy, etc.), blank cluster (IL-13, pathogenesis, epithelial cells, etc.), blue cluster (COPD, severe asthma, rhinovirus, etc.), green cluster (biomarker, IGE, dendritic cells, etc.), yellow cluster (eosinophilia, IL-37, alarmin, etc.), and red cluster (prognosis, IL-25, basophil, etc.).These indicate that keywords in the same cluster are more important research hotspots during the same period.

**Figure 9:**
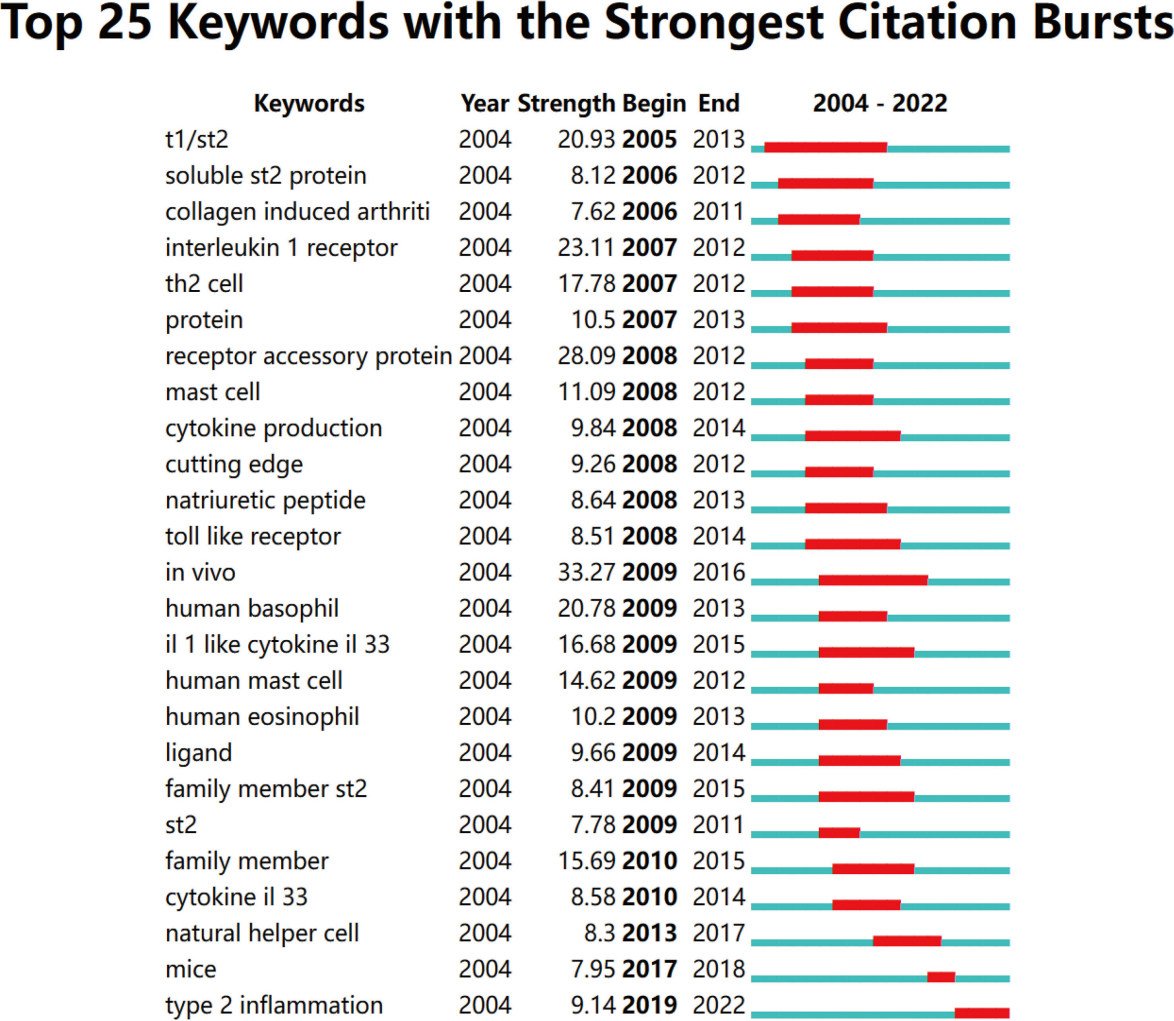
The top 25 keywords with the strongest citation bursts.

Keywords with strong citation bursts are another critical indicator of hotspots and emerging trends in a research field. As shown in **Figure 9**, among the top 25 keywords with the strongest citation bursts, *in vivo* had the strongest burst (33.27), followed by receptor accessory protein (28.09), IL-1r (23.11), T1/ST2 (20.93), and human basophil (20.78). Of note, only Type 2 inflammation (9.14) is still in the burst.

## 4 Discussion

### 4.1 General information

Based on the data from the WoSCC database at December 7, 2022, a total of 4711 articles on IL-33 research have been published in 1009 academic journals by 24652 authors from 483 institutions in 89 countries. Since IL-33 was first reported in 2005(Schmitz et al., 2005), research on IL-33 has begun. This field was still in its infancy in 2004-2006 and lacked a research base. Since 2007 publications related to IL-33 have been steadily and rapidly increasing, with six times the number of publications in 2021 compared to 2011. This means that IL-33 is one of the more popular research areas and has attracted a great deal of scholarly attention.

In the country visualization analysis, the USA and China are the most prolific countries in this area, which is closely linked to the support of local institutions, with 5 of the top 10 institutions also in China and 2 in the USA. The co-occurrence density of 0.11 for countries suggests that IL-33 research is more collaborative worldwide, which is beneficial for the long-term development of academic research. Meanwhile, countries with high-centrality nodes play a key bridging role in the global collaborative network in this field (31, 37), with the USA, in particular, being a central leader in IL-33 research. In addition, the three research institutions that show high centrality are Glasgow University from the UK, which is the most central, followed by Brigham and Women’s Hospital from the USA, indicating a dominant position in the global IL-33 research collaboration.

According to **Table 3** and **Figure 4A**, Frontiers in Immunology published the most IL-33 researches, far more than any other journal but was not among the top 10 co-cited journals. The Journal of Immunology and the Journal of Allergy and Clinical Immunology are the top five most published and top five co-cited journals. These two academic journals have played a crucial role in IL-33 research. Seven of the top 10 prolific journals are among high IF journals. The co-cited journals are primarily high-impact journals, indicating the high value of IL-33 research in the global academic landscape. These popular journals are closely related to cell biology and immunology, which is rather similar to the analysis of the journal dual-map overlay. Most of the existing research is focused on basic research, but there has been a trend toward clinical aspects.

From the author’s perspective, Andrew N. J. McKenzie from Cambridge, UK, has published the most articles and citations on IL-33. Jochen Schmitz from the USA is ranked first among co-cited authors, showing his pre-eminence in the IL-33 research field. Also in the top 5 published and co-cited authors is Foo Yew Liew from the University of Glasgow, England, which explored IL-33 as a universal and important immunomodulator from multiple perspectives. He and his team have also proposed a relationship between IL-33 and diseases such as sepsis (38), oncological chemotherapy (39), Alzheimer’s disease (40), and cerebral malaria (41, 42), and suggest that IL-33 is a double-edged sword, the rational regulation of which may have considerable potential therapeutic effects.

According to **Table 5**, the 15 most cited references are mainly on immune mechanisms about IL-33, the IL-1 family, ILCs, asthma, and others, including four reviews. Of supreme centrality is E Suzanne Cohen, who proposed a mechanism for quick cessation of IL-33 action at its receptor ST2 by conceiving a switch controlled by oxidation and bridging of the free cysteines in IL-33. This article also suggested that cysteine oxidation could play a role in the regulation of many IL-1 family cytokines (33). References with citation bursts mean that the results of studies in this area have been cited often for a period of time. This suggests that the articles have received a lot of interest from the scientific community and may, in part, reflect dynamic shifts and hotspots in IL-33 research. The earliest burst began in 2007, with an article published in 2005, and the burst lasted for four years. It is obvious that four references’ burst is still ongoing, all of which are reviews. They provide a comprehensive and integrated discussion of various aspects of molecular biology, signaling, regulatory mechanisms, and their relationship to the disease of IL-33.

### 4.2 The hotspots and trends

One of the most important roles of bibliometrics is to process and analyze a large amount of data to provide researchers with information on research trends (43). The analysis of frequently recurring keywords might indicate changing trends and prominent subjects, which is important for understanding the evolution of this academic field (44). Before the specific analysis, let’s have a general idea about the evolution of the keywords of IL-33 during the period 2004-2022. As shown in **Figure 8A**, in the early stages of IL-33 research, the main focus was on the fundamental properties of IL-33 biology, and its role in apoptosis, innate immunity, inflammation, and allergic reactions was initially explored. In 2008, the highly significant ST2 (the receptor for IL-33) and its associated signaling became popular. IL-33 also began to be studied in the cardiovascular system and in autoimmune diseases. Subsequently, IL-33-induced activation of related cytokines (e.g., IL-4, IL-13) and their mechanisms of interaction have been extensively studied. The mechanism of IL-33 action on various types of cells (e.g., endothelial cells, macrophages) was also studied in depth. In 2015-2020, IL-33 is repeatedly mentioned and studied for its role in various diseases. In the last three years, type 2 inflammation and type 2 immunity have been the most popularly studied. There is also IL-33 immunotherapy and related tumor microenvironment. It is found that current IL-33 research focuses on immunology, molecular cell biology, and medical research. Meanwhile, the main keywords revolve around molecular biology, immune action and related diseases (**Figure 7A**).

#### 4.2.1 Molecular biology of IL-33

Since IL-33 proposed in 2005, a more comprehensive understanding of the IL-33 gene was available (3). The IL-33 gene consists of eight exons (3). The promoter of the human IL-33 mRNA form is located upstream of the untranslated exon. It has an interferon-stimulated response element (ISRE) and IFN-γ activation site (GAS) (45). Asthma is one of the popular keywords for IL-33 (**Table 6**). It has been a popular research topic, while many of the single nucleotide polymorphisms (SNPs) in the human IL - 33 gene associated with asthma are located in promoters and introns-1 (46), and SNPs in IL-33 are often associated with increased asthma susceptibility (47, 48).

**Table 6:** The top 20 keywords in terms of frequency of occurrence concerning IL-33.

Rank	Keyword	Occurrences	Rank	Keyword	Occurrences
1	interleukin 33	1512	11	cardiovascular diseases	144
2	inflammatory	560	12	interleukin 1	140
3	asthma	479	13	eosinophils	123
4	cytokines	409	14	biomarkers	120
5	soluble suppression of tumorigenicity-2	381	15	th2 cells	108
6	ilcs	277	16	innate immunity	103
7	allergic inflammation	248	17	macrophages	95
8	immunology	173	18	interleukin-25	94
9	allergy	171	19	epithelium	87
10	mast cells	148	20	tslp	81

In this study, the keyword #7 cytokine cluster was highly prominent from 2004-2010, and most publications in the early nascent phase focused on the basic structure and mechanism of IL-33. IL-33 is a tissue-derived cytokine. It was found to be abundant in endothelial cells, tissue epithelial cells and stromal cells in 2008 (4). In the course of experimental studies, species-specific differences in IL-33 were found, with IL-33 being expressed by type II alveolar cells in mice (19, 49). Therefore, the results of mouse models cannot be directly extrapolated to target IL-33 in humans. Mélanie Pichery et al. also experimentally, IL-33 is a nuclear cytokine *in vivo (* 50). Epithelial-derived IL-33 plays a key role in allergic inflammation and type 2 immunity. IL-33 can also be induced to be expressed and further increased in response to cellular stress or inflammation, such as in the intestinal epithelium of bone marrow transplant recipients with GVHD (51), asthma or COPD (17, 19). In addition, immune cells (e.g., macrophages, mast cells, DCs) have been widely studied in 2010 and are considered to be an important cellular source of IL-33 protein *in vivo* (52). However, previous studies claiming IL-33 expression in macrophages did not have proper controls (IL-33 KD/KO cells) (53).

The nuclear localization sequence of IL-33 N-terminal structural domain permits nuclear localization and chromatin binding, and the C-terminal region of the protein has a brief chromatin-binding sequence (3, 25). In 2018, Jared Travers proposed that chromatin binding is a post-translational mechanism that controls IL-33 release and ST2-mediated bioactivity, elaborating on the functional importance of IL-33 nuclear localization and chromatin binding (54). Meanwhile, evidence from many studies suggests that nuclear IL-33 can mediate gene regulation through multiple mechanisms and has transcriptional regulatory functions (55–57). At this time, we are still unsure of the exactly how IL-33 leaves the nucleus and travels outside the cell. Li-Yin Hung et al. proposed that cCDs release IL-33 through a specific plasma membrane conduit (58). Another recent clinical study showed that the truncated IL-33 isoform may be able to regulate secretion on the surface of small extracellular vesicles or exosomes (59). In 2005, IL-33 was considered an inactive cytokine prerequisite that required caspase-1/inflammasomes activation to be biologically active (3). However, it was later shown that IL-33 itself is biologically active and can induce ST2-dependent responses in target cells (34, 60). In 2007, apoptosis-related studies were carried out extensively. During apoptosis, cleavage of IL-33 protein by caspases leads to its inactivation (34). Protein degradation may be an important mechanism limiting the biological activity of IL-33 *in vivo (* 61). Extracellular oxidation of the protein may also be important in terminating the biological activity of IL-33 (33).

The receptor for IL-33, ST2, was identified as an mRNA produced by fibroblasts back in 1989. sST2 is produced from a single mRNA by differential expression and alternative splicing of two distinct promoters (62, 63), and sST2 is also ranked in the top five keywords (**Table 6**). IL-33 signaling *via* ST2 has essential implications for the immune system, which also involves multifaceted regulation (25, 26). Tengfang Li et al. found that when stimulated on macrophages, TLR4 and ST2 exerted different effects on cellular metabolism (64).

#### 4.2.2 The role of IL-33 in immunity

Immunity is the most intensive area of IL-33 research, and its various immunomodulatory mechanisms have remained a hot research topic until now. The effect of IL-33 on type 2 immunity has been the focus of research since 2007. IL-33, IL-25, and TSLP are the innate cytokines that initiate the type 2 response (65). IL-25 and TSLP can synergize with IL-33 to activate ILC2 (11, 66). In 2019, Madelene W Dahlgren identified a new feedback function for IL-33 following helminth infection. IL-33 and TSLP from fibroblast-like adventitial stromal cells(ASCs) are required for intrapulmonary accumulation of ILC2 and Th2 cells during helminth infection, and ILC2 promotes amplification of ASCs and production of IL-33 (67). Meanwhile, IL-33 increases the proliferation of CD8^+^ T cells as well as the production of the type 1 cytokine IFN-γ and TNF-α during infection (16). In 2015, Claudia Baumann proposed that induction of ST2 upregulation in response to Th1 after LCMV infection is dependent on STAT4 and the type 1-associated transcription factor T-BET (68). At the same time, a recent article proposed that during LCMV infection, FRC deficiency in IL-33 protein expression through an unknown mechanism is associated with the induction of ST2 expression and expansion in CD8^+^ T cells (69).

IL-33 enhances macrophage cytokines secretion and CXCR2 expression in fungal infections to drive neutrophil recruitment and bactericidal capacity (70). A study has confirmed that the balance between IL-33 and IL-1 signaling regulates the immune response during infection (71). IL-33 is released in sepsis to activate neutrophils and prevent bacterial growth while repairing tissue (72), but high IL-33 levels can lead to immunosuppression (73). High levels of IL-33 activate ILC2, which produces IL-4 and IL-13, and promote M2 macrophages polarization, hence increasing Treg cells proliferation (38). In sepsis, the induced production of IL-33 represents a trade-off between acute protection and distant immunosuppression.

#### 4.2.3 IL-33 in various diseases

The association between IL-33 and various diseases has been a popular research area since its early days. Disease research has involved inflammatory diseases, tumors, infectious diseases, and central nervous system disorders. The following discussion will focus on inflammatory diseases and cancers.

Inflammatory diseases involve several organ systems, mainly the respiratory, cardiovascular, and gastrointestinal systems. #0 (thymic stromal lymphopoietin) in **Figure 7B** shows that airway inflammation has attracted scholarly attention since 2007 and has remained a popular research topic in recent years (**Figure 8**). IL-33 targets ILC2 and Th2 cells to produce type 2 cytokines and promote type 2 inflammation by activating basophils and mast cells (74–76). IL-33 expression is often upregulated in allergic and respiratory diseases such as asthma, chronic obstructive pulmonary disease, and allergic rhinitis. Another gene-related research presented that IL-33 is related to asthma and that both IL-33 and IL1RL1 are linked to disease susceptibility (77). Yi et al. have identified intelectin (ITLN) knockdown suppressed expression of IL-33, IL-25, and TSLP expression in asthma and atopic dermatitis models (78). IL-33 is associated with IL-9 signaling in mast cells (79). IL-9 is a downstream cytokine associated with the role of IL-33 in the asthmatic airways (80). A study on a mouse model of allergic conjunctivitis found that IL-33 activated CD4^+^ T cells produce IL-9 (81). Meanwhile, the activation of the IL-33/ST2-involving Th2/IL-31 immune response has an important role in allergic inflammation (82). In asthmatics, both Th1 and Th2 responses are associated with the expression of IL-31 and IL-33. The activation of Th2 cells is closely associated with the pathogenic effects of IL-33, ultimately leading to inflammation (82). Airway inflammation is often accompanied by airway remodeling (83). IL-33 also plays an essential role in the pathological process of airway remodeling (84).

In 2004, Masahisa Shimpo et al. suggested that sST2 concentration was linked to damaged left ventricular function and a poor prognosis (21). It was demonstrated that IL-33 could reduce hypertrophy and fibrosis in mouse ventricles, and infusion of sST2 could antagonize the antihypertrophic effect of IL-33 (20). Nonetheless, studies on the clinical application of the IL-33-ST2 remain at the stage of using sST2 as a prognostic marker for myocardial infarction.

Elevated IL-33 levels in IBD patients correlate with disease severity, and IL-33 may serve as a potential biomarker for IBD (85). A study found that IL-33 promoted Th2 and Treg cell responses to ameliorate colitis induced by trinitrobenzene sulfonic acid (TNBS) in mice in a Foxp3-dependent form (86). IL-33 was found to enhance the expansion of Foxp3^+^ Treg cells in the intestine *via* transforming growth factor-β (TGF-β), thereby suppressing the intestinal inflammatory response (87). IL-33 could also affect the activation of inflammatory response-associated macrophages through T-cell differentiation effects (88). In addition, IL-33 modulates the immune inflammatory reaction to the physical and biological barriers of the intestine (89). However, the impact of ST2-specific expression in the intestinal mucosa of IBD on IL-33 as well as on disease progression remains unclear.

According to **Figures 7B**, **8A**, cancer is a relatively recent disease to be studied concerning IL-33 and has still been a buzzword. IL-33 is engaged in the initiation and progression of many cancers like lung cancer (90), gastric cancer (91), bile duct cancer (92), breast cancer (93), and multiple myeloma (94). Mast cells are activated by tumor-derived IL-33 through the recruitment of tumor-associated macrophages and their support of vascular networks to maintain tumor growth (95). The secretion of reparative growth factors, such as Areg, by IL-33-stimulated ST2^+^ Tregs increased metastatic mammary carcinoma in a mouse model (96). Also, IL-33 can restrict tumor growth and metastasis through eosinophils (97). Marek Wagner et al. found that tumor-derived lactate attenuates the function and survival of ILC2, thereby disrupting the IL-33/ILC2/eosinophil axis function (98). A research reported that infusion of IL-33 is effective in suppressing lung metastases from mammary carcinoma in mice, which may be related to the elevation of NK cells at TME (99). In contrast, it was previously reported that mice lacking ST2 inhibited breast cancer development by enhancing the cytotoxic activity of NK cells (100). This contradictory result deserves an in-depth study. The combination of IL-33 and immune checkpoint blockade(ICB) has a better anti-tumor effect (101). In recent years, many studies have reported that IL-33, eosinophils in TME can better perform the efficacy of anti-PD1/anti-CTLA-4 therapies (102, 103). Daniel O Villarreal et al. showed that IL-33 can be used as an immune adjuvant for cancer vaccination (101). Treatment with IL-33 also drives the cytotoxic activities of Tc9 cells induced by DCs thereby promoting the therapeutic efficiency of tumor vaccines based on DCs (104). Notably, IL-33 is a pleiotropic cytokine with both pro- and anti-tumor effects, and its rational use in cancer immunotherapy needs to be extensively studied.

### 4.3 Limitations

In this study, there are still some limitations. Firstly, all of the data were obtained from the WoSCC. Even though WoSCC contains the majority of the publications, some publications are likely not included into this analysis. Furthermore, the quality of the articles collected for this study varies, thereby undermining the credibility of the analysis. Finally, CiteSpace and VOSviewer themselves have inherent limitations. Terms extracted from literature titles, abstracts, and keywords may exhibit a high degree of variability during cluster analysis, and there is no guarantee that all terms with the same meaning will be combined when combining terms with the same meaning.

## 5 Conclusion

In summary, IL-33 research continues to develop steadily worldwide. The USA and China are prominent contributors to the IL-33 research field. Frontiers in Immunology and the Journal of Immunology are among the more influential journals in this field of research. Jochen Schmitz from the USA has made an outstanding contribution to IL-33 research. Immunology and molecular biology of IL-33 are currently hot research areas. Due to the dual role of IL-33, there is a current trend to investigate the immune mechanisms of IL-33 in various diseases, which may be a potential therapeutic target to be developed for disease treatments. Hence, this bibliometric analysis may provide an objective perspective on IL-33 and help scholars to track knowledge and research directions of IL-33.

## Data availability statement

The original contributions presented in the study are included in the article/supplementary material. Further inquiries can be directed to the corresponding authors.

## Author contributions

DL and JL concepted this study. JJ, YW, and QS designed this study. JJ and YW were involved in the data collection and analysis. YW normalized the pictures. JJ wrote the manuscript. DL, JL, JJ, YW, and QS revised and approved the final version of the manuscript. All authors contributed to the article and approved the submitted version.

## References

[1] Onda H Kasuya H Takakura K Hori T Imaizumi T Takeuchi T. Identification of genes differentially expressed in canine vasospastic cerebral arteries after subarachnoid hemorrhage. J Cereb Blood Flow Metabol: Off J Int Soc Cereb Blood Flow Metab (1999) 19(11):1279–88. doi: 10.1097/00004647-199911000-00013 10566975

[2] Baekkevold ES Roussigné M Yamanaka T Johansen FE Jahnsen FL Amalric F. Molecular characterization of nf-hev, a nuclear factor preferentially expressed in human high endothelial venules. Am J Pathol (2003) 163(1):69–79. doi: 10.1016/s0002-9440(10)63631-0 12819012 PMC1868188

[3] Schmitz J Owyang A Oldham E Song Y Murphy E McClanahan TK. Il-33, an interleukin-1-Like cytokine that signals *Via* the il-1 receptor-related protein St2 and induces T helper type 2-associated cytokines. Immunity (2005) 23(5):479–90. doi: 10.1016/j.immuni.2005.09.015 16286016

[4] Moussion C Ortega N Girard JP. The il-1-Like cytokine il-33 is constitutively expressed in the nucleus of endothelial cells and epithelial cells in vivo: A novel ‘Alarmin’? PloS One (2008) 3(10):e3331. doi: 10.1371/journal.pone.0003331 18836528 PMC2556082

[5] Lingel A Weiss TM Niebuhr M Pan B Appleton BA Wiesmann C. Structure of il-33 and its interaction with the St2 and il-1racp receptors–insight into heterotrimeric il-1 signaling complexes. Structure (London England: 1993) (2009) 17(10):1398–410. doi: 10.1016/j.str.2009.08.009 PMC2766095 19836339

[6] Cayrol C Girard JP. The il-1-Like cytokine il-33 is inactivated after maturation by caspase-1. Proc Natl Acad Sci USA (2009) 106(22):9021–6. doi: 10.1073/pnas.0812690106 PMC2690027 19439663

[7] Cayrol C Girard JP. Il-33: An alarmin cytokine with crucial roles in innate immunity, inflammation and allergy. Curr Opin Immunol (2014) 31:31–7. doi: 10.1016/j.coi.2014.09.004 25278425

[8] Spits H Cupedo T. Innate lymphoid cells: Emerging insights in development, lineage relationships, and function. Annu Rev Immunol (2012) 30:647–75. doi: 10.1146/annurev-immunol-020711-075053 22224763

[9] Scanlon ST McKenzie AN. Type 2 innate lymphoid cells: New players in asthma and allergy. Curr Opin Immunol (2012) 24(6):707–12. doi: 10.1016/j.coi.2012.08.009 22985480

[10] Moro K Yamada T Tanabe M Takeuchi T Ikawa T Kawamoto H. Innate production of T(H)2 cytokines by adipose tissue-associated c-Kit(+)Sca-1(+) lymphoid cells. Nature (2010) 463(7280):540–4. doi: 10.1038/nature08636 20023630

[11] Neill DR Wong SH Bellosi A Flynn RJ Daly M Langford TK. Nuocytes represent a new innate effector leukocyte that mediates type-2 immunity. Nature (2010) 464(7293):1367–70. doi: 10.1038/nature08900 PMC2862165 20200518

[12] Price AE Liang HE Sullivan BM Reinhardt RL Eisley CJ Erle DJ. Systemically dispersed innate il-13-Expressing cells in type 2 immunity. Proc Natl Acad Sci USA (2010) 107(25):11489–94. doi: 10.1073/pnas.1003988107 PMC2895098 20534524

[13] Löhning M Stroehmann A Coyle AJ Grogan JL Lin S Gutierrez-Ramos JC. T1/St2 is preferentially expressed on murine Th2 cells, independent of interleukin 4, interleukin 5, and interleukin 10, and important for Th2 effector function. Proc Natl Acad Sci USA (1998) 95(12):6930–5. doi: 10.1073/pnas.95.12.6930 PMC22690 9618516

[14] Trajkovic V Sweet MJ Xu D. T1/St2–an il-1 receptor-like modulator of immune responses. Cytokine Growth Factor Rev (2004) 15(2-3):87–95. doi: 10.1016/j.cytogfr.2004.02.004 15110792

[15] Smithgall MD Comeau MR Yoon BR Kaufman D Armitage R Smith DE. Il-33 amplifies both Th1- and Th2-type responses through its activity on human basophils, allergen-reactive Th2 cells, inkt and nk cells. Int Immunol (2008) 20(8):1019–30. doi: 10.1093/intimm/dxn060 18550585

[16] Bonilla WV Fröhlich A Senn K Kallert S Fernandez M Johnson S. The alarmin interleukin-33 drives protective antiviral Cd8^+^ T cell responses. Sci (New York NY) (2012) 335(6071):984–9. doi: 10.1126/science.1215418 22323740

[17] Préfontaine D Lajoie-Kadoch S Foley S Audusseau S Olivenstein R Halayko AJ. Increased expression of il-33 in severe asthma: Evidence of expression by airway smooth muscle cells. J Immunol (Baltimore Md: 1950) (2009) 183(8):5094–103. doi: 10.4049/jimmunol.0802387 19801525

[18] Sedhom MA Pichery M Murdoch JR Foligné B Ortega N Normand S. Neutralisation of the interleukin-33/St2 pathway ameliorates experimental colitis through enhancement of mucosal healing in mice. Gut (2013) 62(12):1714–23. doi: 10.1136/gutjnl-2011-301785 PMC3841767 23172891

[19] Kearley J Silver JS Sanden C Liu Z Berlin AA White N. Cigarette smoke silences innate lymphoid cell function and facilitates an exacerbated type I interleukin-33-Dependent response to infection. Immunity (2015) 42(3):566–79. doi: 10.1016/j.immuni.2015.02.011 25786179

[20] Sanada S Hakuno D Higgins LJ Schreiter ER McKenzie AN Lee RT. Il-33 and St2 comprise a critical biomechanically induced and cardioprotective signaling system. J Clin Invest (2007) 117(6):1538–49. doi: 10.1172/jci30634 PMC1865027 17492053

[21] Shimpo M Morrow DA Weinberg EO Sabatine MS Murphy SA Antman EM. Serum levels of the interleukin-1 receptor family member St2 predict mortality and clinical outcome in acute myocardial infarction. Circulation (2004) 109(18):2186–90. doi: 10.1161/01.Cir.0000127958.21003.5a 15117853

[22] Savinko T Matikainen S Saarialho-Kere U Lehto M Wang G Lehtimäki S. Il-33 and St2 in atopic dermatitis: Expression profiles and modulation by triggering factors. J Invest Dermatol (2012) 132(5):1392–400. doi: 10.1038/jid.2011.446 22277940

[23] Maywald RL Doerner SK Pastorelli L De Salvo C Benton SM Dawson EP. Il-33 activates tumor stroma to promote intestinal polyposis. Proc Natl Acad Sci USA (2015) 112(19):E2487–96. doi: 10.1073/pnas.1422445112 PMC4434739 25918379

[24] Mager LF Riether C Schürch CM Banz Y Wasmer MH Stuber R. Il-33 signaling contributes to the pathogenesis of myeloproliferative neoplasms. J Clin Invest (2015) 125(7):2579–91. doi: 10.1172/jci77347 PMC4563674 26011644

[25] Cayrol C Girard JP. Interleukin-33 (Il-33): A nuclear cytokine from the il-1 family. Immunol Rev (2018) 281(1):154–68. doi: 10.1111/imr.12619 29247993

[26] Liew FY Girard JP Turnquist HR. Interleukin-33 in health and disease. Nat Rev Immunol (2016) 16(11):676–89. doi: 10.1038/nri.2016.95 27640624

[27] Smith DR. Bibliometrics, dermatology and contact dermatitis. Contact Dermatitis (2008) 59(3):133–6. doi: 10.1111/j.1600-0536.2008.01405.x 18759892

[28] Ma C Su H Li H. Global research trends on prostate diseases and erectile dysfunction: A bibliometric and visualized study. Front Oncol (2020) 10:627891. doi: 10.3389/fonc.2020.627891 33643922 PMC7908828

[29] Ke L Lu C Shen R Lu T Ma B Hua Y. Knowledge mapping of drug-induced liver injury: A scientometric investigation (2010-2019). Front Pharmacol (2020) 11:842. doi: 10.3389/fphar.2020.00842 32581801 PMC7291871

[30] Zhang J Song L Xu L Fan Y Wang T Tian W. Knowledge domain and emerging trends in ferroptosis research: A bibliometric and knowledge-map analysis. Front Oncol (2021) 11:686726. doi: 10.3389/fonc.2021.686726 34150654 PMC8209495

[31] Chen C. Searching for intellectual turning points: Progressive knowledge domain visualization. Proc Natl Acad Sci USA (2004) 101 Suppl 1(Suppl 1):5303–10. doi: 10.1073/pnas.0307513100 PMC387312 14724295

[32] van Eck NJ Waltman L. Software survey: Vosviewer, a computer program for bibliometric mapping. Scientometrics (2010) 84(2):523–38. doi: 10.1007/s11192-009-0146-3 PMC2883932 20585380

[33] Cohen ES Scott IC Majithiya JB Rapley L Kemp BP England E. Oxidation of the alarmin il-33 regulates St2-dependent inflammation. Nat Commun (2015) 6:8327. doi: 10.1038/ncomms9327 26365875 PMC4579851

[34] Lüthi AU Cullen SP McNeela EA Duriez PJ Afonina IS Sheridan C. Suppression of interleukin-33 bioactivity through proteolysis by apoptotic caspases. Immunity (2009) 31(1):84–98. doi: 10.1016/j.immuni.2009.05.007 19559631

[35] Madouri F Guillou N Fauconnier L Marchiol T Rouxel N Chenuet P. Caspase-1 activation by Nlrp3 inflammasome dampens il-33-Dependent house dust mite-induced allergic lung inflammation. J Mol Cell Biol (2015) 7(4):351–65. doi: 10.1093/jmcb/mjv012 25714839

[36] Palmer G Lipsky BP Smithgall MD Meininger D Siu S Talabot-Ayer D. The il-1 receptor accessory protein (Acp) is required for il-33 signaling and soluble acp enhances the ability of soluble St2 to inhibit il-33. Cytokine (2008) 42(3):358–64. doi: 10.1016/j.cyto.2008.03.008 18450470

[37] Liu S Xia K Liu X Duan Y Hu M Xia H. Bibliometric analysis of birt-Hogg-Dubé syndrome from 2001 to 2021. Front Med (2022) 9:857127. doi: 10.3389/fmed.2022.857127 PMC9035795 35479937

[38] Nascimento DC Melo PH Piñeros AR Ferreira RG Colón DF Donate PB. Il-33 contributes to sepsis-induced long-term immunosuppression by expanding the regulatory T cell population. Nat Commun (2017) 8:14919. doi: 10.1038/ncomms14919 28374774 PMC5382289

[39] Guabiraba R Besnard AG Menezes GB Secher T Jabir MS Amaral SS. Il-33 targeting attenuates intestinal mucositis and enhances effective tumor chemotherapy in mice. Mucosal Immunol (2014) 7(5):1079–93. doi: 10.1038/mi.2013.124 PMC4077764 24424522

[40] Fu AK Hung KW Yuen MY Zhou X Mak DS Chan IC. Il-33 ameliorates alzheimer’s disease-like pathology and cognitive decline. Proc Natl Acad Sci USA (2016) 113(19):E2705–13. doi: 10.1073/pnas.1604032113 PMC4868478 27091974

[41] Besnard AG Guabiraba R Niedbala W Palomo J Reverchon F Shaw TN. Il-33-Mediated protection against experimental cerebral malaria is linked to induction of type 2 innate lymphoid cells, M2 macrophages and regulatory T cells. PloS Pathog (2015) 11(2):e1004607. doi: 10.1371/journal.ppat.1004607 25659095 PMC4450060

[42] Strangward P Haley MJ Albornoz MG Barrington J Shaw T Dookie R. Targeting the Il33-Nlrp3 axis improves therapy for experimental cerebral malaria. Proc Natl Acad Sci USA (2018) 115(28):7404–9. doi: 10.1073/pnas.1801737115 PMC6048513 29954866

[43] Xiao F Li C Sun J Zhang L. Knowledge domain and emerging trends in organic photovoltaic technology: A scientometric review based on citespace analysis. Front Chem (2017) 5:67. doi: 10.3389/fchem.2017.00067 28966923 PMC5605557

[44] Wang Y Jia Y Li M Jiao S Zhao H. Hotspot and frontier analysis of exercise training therapy for heart failure complicated with depression based on web of science database and big data analysis. Front Cardiovasc Med (2021) 8:665993. doi: 10.3389/fcvm.2021.665993 34095256 PMC8169975

[45] Tsuda H Komine M Tominaga SI Ohtsuki M. Identification of the promoter region of human il-33 responsive to induction by ifnγ. J Dermatol Sci (2017) 85(2):137–40. doi: 10.1016/j.jdermsci.2016.11.002 27955843

[46] Grotenboer NS Ketelaar ME Koppelman GH Nawijn MC. Decoding asthma: Translating genetic variation in Il33 and Il1rl1 into disease pathophysiology. J Allergy Clin Immunol (2013) 131(3):856–65. doi: 10.1016/j.jaci.2012.11.028 23380221

[47] Gudbjartsson DF Bjornsdottir US Halapi E Helgadottir A Sulem P Jonsdottir GM. Sequence variants affecting eosinophil numbers associate with asthma and myocardial infarction. Nat Genet (2009) 41(3):342–7. doi: 10.1038/ng.323 19198610

[48] Moffatt MF Gut IG Demenais F Strachan DP Bouzigon E Heath S. A Large-scale, consortium-based genomewide association study of asthma. New Engl J Med (2010) 363(13):1211–21. doi: 10.1056/NEJMoa0906312 PMC4260321 20860503

[49] Hardman CS Panova V McKenzie AN. Il-33 citrine reporter mice reveal the temporal and spatial expression of il-33 during allergic lung inflammation. Eur J Immunol (2013) 43(2):488–98. doi: 10.1002/eji.201242863 PMC3734634 23169007

[50] Pichery M Mirey E Mercier P Lefrancais E Dujardin A Ortega N. Endogenous il-33 is highly expressed in mouse epithelial barrier tissues, lymphoid organs, brain, embryos, and inflamed tissues: *In situ* analysis using a novel il-33-Lacz gene trap reporter strain. J Immunol (Baltimore Md: 1950) (2012) 188(7):3488–95. doi: 10.4049/jimmunol.1101977 22371395

[51] Reichenbach DK Schwarze V Matta BM Tkachev V Lieberknecht E Liu Q. The il-33/St2 axis augments effector T-cell responses during acute gvhd. Blood (2015) 125(20):3183–92. doi: 10.1182/blood-2014-10-606830 PMC4432012 25814531

[52] Chang YJ Kim HY Albacker LA Baumgarth N McKenzie AN Smith DE. Innate lymphoid cells mediate influenza-induced airway hyper-reactivity independently of adaptive immunity. Nat Immunol (2011) 12(7):631–8. doi: 10.1038/ni.2045 PMC3417123 21623379

[53] Cayrol C Girard JP. Interleukin-33 (Il-33): A critical review of its biology and the mechanisms involved in its release as a potent extracellular cytokine. Cytokine (2022) 156:155891. doi: 10.1016/j.cyto.2022.155891 35640416

[54] Travers J Rochman M Miracle CE Habel JE Brusilovsky M Caldwell JM. Chromatin regulates il-33 release and extracellular cytokine activity. Nat Commun (2018) 9(1):3244. doi: 10.1038/s41467-018-05485-x 30108214 PMC6092330

[55] Ali S Mohs A Thomas M Klare J Ross R Schmitz ML. The dual function cytokine il-33 interacts with the transcription factor nf-κb to dampen nf-κb-Stimulated gene transcription. J Immunol (Baltimore Md: 1950) (2011) 187(4):1609–16. doi: 10.4049/jimmunol.1003080 21734074

[56] Gatti F Mia S Hammarström C Frerker N Fosby B Wang J. Nuclear il-33 restrains the early conversion of fibroblasts to an extracellular matrix-secreting phenotype. Sci Rep (2021) 11(1):108. doi: 10.1038/s41598-020-80509-5 33420328 PMC7794291

[57] Tominaga S. A putative protein of a growth specific cdna from Balb/C-3t3 cells is highly similar to the extracellular portion of mouse interleukin 1 receptor. FEBS Lett (1989) 258(2):301–4. doi: 10.1016/0014-5793(89)81679-5 2532153

[58] Hung LY Tanaka Y Herbine K Pastore C Singh B Ferguson A. Cellular context of il-33 expression dictates impact on anti-helminth immunity. Sci Immunol (2020) 5(53):eabc6259. doi: 10.1126/sciimmunol.abc6259 33188058 PMC8257082

[59] Katz-Kiriakos E Steinberg DF Kluender CE Osorio OA Newsom-Stewart C Baronia A. Epithelial il-33 appropriates exosome trafficking for secretion in chronic airway disease. JCI Insight (2021) 6(4):e136166. doi: 10.1172/jci.insight.136166 33507882 PMC7934940

[60] Cayrol C Duval A Schmitt P Roga S Camus M Stella A. Environmental allergens induce allergic inflammation through proteolytic maturation of il-33. Nat Immunol (2018) 19(4):375–85. doi: 10.1038/s41590-018-0067-5 29556000

[61] Kouzaki H Iijima K Kobayashi T O’Grady SM Kita H. The danger signal, extracellular atp, is a sensor for an airborne allergen and triggers il-33 release and innate Th2-type responses. J Immunol (Baltimore Md: 1950) (2011) 186(7):4375–87. doi: 10.4049/jimmunol.1003020 PMC3062674 21357533

[62] Yanagisawa K Takagi T Tsukamoto T Tetsuka T Tominaga S. Presence of a novel primary response gene St2l, encoding a product highly similar to the interleukin 1 receptor type 1. FEBS Lett (1993) 318(1):83–7. doi: 10.1016/0014-5793(93)81333-u 7916701

[63] Lipsky BP Toy DY Swart DA Smithgall MD Smith D. Deletion of the St2 proximal promoter disrupts fibroblast-specific expression but does not reduce the amount of soluble St2 in circulation. Eur J Immunol (2012) 42(7):1863–9. doi: 10.1002/eji.201142274 22585662

[64] Li T Zhang Z Bartolacci JG Dwyer GK Liu Q Mathews LR. Graft il-33 regulates infiltrating macrophages to protect against chronic rejection. J Clin Invest (2020) 130(10):5397–412. doi: 10.1172/jci133008 PMC7524467 32644975

[65] Palmieri V Ebel JF Ngo Thi Phuong N Klopfleisch R Vu VP Adamczyk A. Interleukin-33 signaling exacerbates experimental infectious colitis by enhancing gut permeability and inhibiting protective Th17 immunity. Mucosal Immunol (2021) 14(4):923–36. doi: 10.1038/s41385-021-00386-7 PMC8221996 33654214

[66] Barlow JL Peel S Fox J Panova V Hardman CS Camelo A. Il-33 is more potent than il-25 in provoking il-13-Producing nuocytes (Type 2 innate lymphoid cells) and airway contraction. J Allergy Clin Immunol (2013) 132(4):933–41. doi: 10.1016/j.jaci.2013.05.012 23810766

[67] Dahlgren MW Jones SW Cautivo KM Dubinin A Ortiz-Carpena JF Farhat S. Adventitial stromal cells define group 2 innate lymphoid cell tissue niches. Immunity (2019) 50(3):707–22.e6. doi: 10.1016/j.immuni.2019.02.002 30824323 PMC6553479

[68] Baumann C Bonilla WV Fröhlich A Helmstetter C Peine M Hegazy AN. T-Bet- and Stat4-dependent il-33 receptor expression directly promotes antiviral Th1 cell responses. Proc Natl Acad Sci USA (2015) 112(13):4056–61. doi: 10.1073/pnas.1418549112 PMC4386370 25829541

[69] Aparicio-Domingo P Cannelle H Buechler MB Nguyen S Kallert SM Favre S. Fibroblast-derived il-33 is dispensable for lymph node homeostasis but critical for Cd8 T-cell responses to acute and chronic viral infection. Eur J Immunol (2021) 51(1):76–90. doi: 10.1002/eji.201948413 32700362

[70] Le HT Tran VG Kim W Kim J Cho HR Kwon B. Il-33 priming regulates multiple steps of the neutrophil-mediated anti-candida albicans response by modulating tlr and dectin-1 signals. J Immunol (Baltimore Md: 1950) (2012) 189(1):287–95. doi: 10.4049/jimmunol.1103564 22661085

[71] Alvarez F Istomine R Shourian M Pavey N Al-Aubodah TA Qureshi S. The alarmins il-1 and il-33 differentially regulate the functional specialisation of Foxp3(+) regulatory T cells during mucosal inflammation. Mucosal Immunol (2019) 12(3):746–60. doi: 10.1038/s41385-019-0153-5 30872761

[72] Alves-Filho JC Sônego F Souto FO Freitas A Verri WA Jr. Auxiliadora-Martins M. Interleukin-33 attenuates sepsis by enhancing neutrophil influx to the site of infection. Nat Med (2010) 16(6):708–12. doi: 10.1038/nm.2156 20473304

[73] Boomer JS To K Chang KC Takasu O Osborne DF Walton AH. Immunosuppression in patients who die of sepsis and multiple organ failure. Jama (2011) 306(23):2594–605. doi: 10.1001/jama.2011.1829 PMC3361243 22187279

[74] Gordon ED Simpson LJ Rios CL Ringel L Lachowicz-Scroggins ME Peters MC. Alternative splicing of interleukin-33 and type 2 inflammation in asthma. Proc Natl Acad Sci USA (2016) 113(31):8765–70. doi: 10.1073/pnas.1601914113 PMC4978244 27432971

[75] Stolarski B Kurowska-Stolarska M Kewin P Xu D Liew FY. Il-33 exacerbates eosinophil-mediated airway inflammation. J Immunol (Baltimore Md: 1950) (2010) 185(6):3472–80. doi: 10.4049/jimmunol.1000730 20693421

[76] Kurowska-Stolarska M Stolarski B Kewin P Murphy G Corrigan CJ Ying S. Il-33 amplifies the polarization of alternatively activated macrophages that contribute to airway inflammation. J Immunol (Baltimore Md: 1950) (2009) 183(10):6469–77. doi: 10.4049/jimmunol.0901575 19841166

[77] Bønnelykke K Sleiman P Nielsen K Kreiner-Møller E Mercader JM Belgrave D. A genome-wide association study identifies Cdhr3 as a susceptibility locus for early childhood asthma with severe exacerbations. Nat Genet (2014) 46(1):51–5. doi: 10.1038/ng.2830 24241537

[78] Yi L Cheng D Zhang K Huo X Mo Y Shi H. Intelectin contributes to allergen-induced il-25, il-33, and tslp expression and type 2 response in asthma and atopic dermatitis. Mucosal Immunol (2017) 10(6):1491–503. doi: 10.1038/mi.2017.10 PMC5568519 28224996

[79] Enoksson M Lyberg K Möller-Westerberg C Fallon PG Nilsson G Lunderius-Andersson C. Mast cells as sensors of cell injury through il-33 recognition. J Immunol (Baltimore Md: 1950) (2011) 186(4):2523–8. doi: 10.4049/jimmunol.1003383 21239713

[80] Du X Li C Wang W Huang Q Wang J Tong Z. Il-33 induced airways inflammation is partially dependent on il-9. Cell Immunol (2020) 352:104098. doi: 10.1016/j.cellimm.2020.104098 32241531

[81] Hu J Gao N Zhang Y Chen X Li J Bian F. Il-33/St2/Il-9/Il-9r signaling disrupts ocular surface barrier in allergic inflammation. Mucosal Immunol (2020) 13(6):919–30. doi: 10.1038/s41385-020-0288-4 PMC7572432 32358573

[82] Murdaca G Greco M Tonacci A Negrini S Borro M Puppo F. Il-33/Il-31 axis in immune-mediated and allergic diseases. Int J Mol Sci (2019) 20(23):5856. doi: 10.3390/ijms20235856 31766607 PMC6929191

[83] Homer RJ Elias JA. Airway remodeling in asthma: Therapeutic implications of mechanisms. Physiol (Bethesda Md) (2005) 20:28–35. doi: 10.1152/physiol.00035.2004 15653837

[84] Ramaprakash H Shibata T Duffy KE Ismailoglu UB Bredernitz RM Moreira AP. Targeting St2l potentiates cpg-mediated therapeutic effects in a chronic fungal asthma model. Am J Pathol (2011) 179(1):104–15. doi: 10.1016/j.ajpath.2011.03.032 PMC3123853 21640974

[85] Pastorelli L Garg RR Hoang SB Spina L Mattioli B Scarpa M. Epithelial-derived il-33 and its receptor St2 are dysregulated in ulcerative colitis and in experimental Th1/Th2 driven enteritis. Proc Natl Acad Sci USA (2010) 107(17):8017–22. doi: 10.1073/pnas.0912678107 PMC2867895 20385815

[86] Duan L Chen J Zhang H Yang H Zhu P Xiong A. Interleukin-33 ameliorates experimental colitis through promoting Th2/Foxp3^+^ regulatory T-cell responses in mice. Mol Med (Cambridge Mass) (2012) 18(1):753–61. doi: 10.2119/molmed.2011.00428 PMC3409280 22426954

[87] Mikulski Z Johnson R Shaked I Kim G Nowyhed H Goodman W. Samp1/Yitfc mice develop ileitis *Via* loss of Ccl21 and defects in dendritic cell migration. Gastroenterology (2015) 148(4):783–93.e5. doi: 10.1053/j.gastro.2015.01.027 25620669 PMC4375031

[88] Beltrán CJ Núñez LE Díaz-Jiménez D Farfan N Candia E Heine C. Characterization of the novel St2/Il-33 system in patients with inflammatory bowel disease. Inflammatory Bowel Dis (2010) 16(7):1097–107. doi: 10.1002/ibd.21175 20014018

[89] Seo DH Che X Kwak MS Kim S Kim JH Ma HW. Interleukin-33 regulates intestinal inflammation by modulating macrophages in inflammatory bowel disease. Sci Rep (2017) 7(1):851. doi: 10.1038/s41598-017-00840-2 28404987 PMC5429815

[90] Kim MS Kim E Heo JS Bae DJ Lee JU Lee TH. Circulating il-33 level is associated with the progression of lung cancer. Lung Cancer (Amsterdam Netherlands) (2015) 90(2):346–51. doi: 10.1016/j.lungcan.2015.08.011 26342550

[91] Sun P Ben Q Tu S Dong W Qi X Wu Y. Serum interleukin-33 levels in patients with gastric cancer. Digest Dis Sci (2011) 56(12):3596–601. doi: 10.1007/s10620-011-1760-5 21643739

[92] Nakagawa H Hayata Y Yamada T Kawamura S Suzuki N Koike K. Peribiliary glands as the cellular origin of biliary tract cancer. Int J Mol Sci (2018) 19(6):1745. doi: 10.3390/ijms19061745 29895797 PMC6032423

[93] Liu J Shen JX Hu JL Huang WH Zhang GJ. Significance of interleukin-33 and its related cytokines in patients with breast cancers. Front Immunol (2014) 5:141. doi: 10.3389/fimmu.2014.00141 24778632 PMC3985005

[94] Musolino C Allegra A Profita M Alonci A Saitta S Russo S. Reduced il-33 plasma levels in multiple myeloma patients are associated with more advanced stage of disease. Br J Haematol (2013) 160(5):709–10. doi: 10.1111/bjh.12146 23205532

[95] Eissmann MF Dijkstra C Jarnicki A Phesse T Brunnberg J Poh AR. Il-33-Mediated mast cell activation promotes gastric cancer through macrophage mobilization. Nat Commun (2019) 10(1):2735. doi: 10.1038/s41467-019-10676-1 31227713 PMC6588585

[96] Halvorsen EC Franks SE Wadsworth BJ Harbourne BT Cederberg RA Steer CA. Il-33 increases St2(+) tregs and promotes metastatic tumour growth in the lungs in an amphiregulin-dependent manner. Oncoimmunology (2019) 8(2):e1527497. doi: 10.1080/2162402x.2018.1527497 30713780 PMC6343789

[97] Andreone S Spadaro F Buccione C Mancini J Tinari A Sestili P. Il-33 promotes Cd11b/Cd18-mediated adhesion of eosinophils to cancer cells and synapse-polarized degranulation leading to tumor cell killing. Cancers (2019) 11(11):1664. doi: 10.3390/cancers11111664 31717819 PMC6895824

[98] Wagner M Ealey KN Tetsu H Kiniwa T Motomura Y Moro K. Tumor-derived lactic acid contributes to the paucity of intratumoral Ilc2s. Cell Rep (2020) 30(8):2743–57.e5. doi: 10.1016/j.celrep.2020.01.103 32101749

[99] Qi L Zhang Q Miao Y Kang W Tian Z Xu D. Interleukin-33 activates and recruits natural killer cells to inhibit pulmonary metastatic cancer development. Int J Cancer (2020) 146(5):1421–34. doi: 10.1002/ijc.32779 31709531

[100] Jovanovic I Radosavljevic G Mitrovic M Juranic VL McKenzie AN Arsenijevic N. St2 deletion enhances innate and acquired immunity to murine mammary carcinoma. Eur J Immunol (2011) 41(7):1902–12. doi: 10.1002/eji.201141417 PMC3746127 21484786

[101] Villarreal DO Wise MC Walters JN Reuschel EL Choi MJ Obeng-Adjei N. Alarmin il-33 acts as an immunoadjuvant to enhance antigen-specific tumor immunity. Cancer Res (2014) 74(6):1789–800. doi: 10.1158/0008-5472.Can-13-2729 PMC4130175 24448242

[102] Hollande C Boussier J Ziai J Nozawa T Bondet V Phung W. Inhibition of the dipeptidyl peptidase Dpp4 (Cd26) reveals il-33-Dependent eosinophil-mediated control of tumor growth. Nat Immunol (2019) 20(3):257–64. doi: 10.1038/s41590-019-0321-5 30778250

[103] Simon SCS Hu X Panten J Grees M Renders S Thomas D. Eosinophil accumulation predicts response to melanoma treatment with immune checkpoint inhibitors. Oncoimmunology (2020) 9(1):1727116. doi: 10.1080/2162402x.2020.1727116 32117594 PMC7028332

[104] Liu N Jiang Y Chen J Nan H Zhao Y Chu X. Il-33 drives the antitumor effects of dendritic cells *Via* the induction of Tc9 cells. Cell Mol Immunol (2019) 16(7):644–51. doi: 10.1038/s41423-018-0166-0 PMC6804534 30275536

